# Distinguishing Between Monomeric scFv and Diabody in Solution Using Light and Small Angle X-ray Scattering

**DOI:** 10.3390/antib8040048

**Published:** 2019-09-23

**Authors:** Frank Lüdel, Sandra Bufe, Willem M. Bleymüller, Hugo de Jonge, Luisa Iamele, Hartmut H. Niemann, Thomas Hellweg

**Affiliations:** 1 Department of Chemistry, Physical and Biophysical Chemistry, Bielefeld University, 33615 Bielefeld, Germany; frank.luedel@mail.de; 2 Department of Chemistry, Structural Biochemistry, Bielefeld University, 33615 Bielefeld, Germany; bufe.sandra@mh-hannover.de (S.B.); willem_manuel.bleymueller@uni-bielefeld.de (W.M.B.); hartmut.niemann@uni-bielefeld.de (H.H.N.); 3 Department of Molecular Medicine, Division of Immunology and General Pathology, University of Pavia, 27100 Pavia, Italy; hugo.dejonge@unipv.it (H.d.J.); luisa.iamele@unipv.it (L.I.)

**Keywords:** DLS, SAXS, SLS, Zimm-Plot, IgG, antibody contstruct

## Abstract

Depending on the linker length between the V${}_{H}$ and the V${}_{L}$ domain, single-chain Fv (scFv) antibody fragments form monomers, dimers (diabodies) or higher oligomers. We aimed at generating a diabody of the anti-MET antibody 3H3 to use it as crystallization chaperone to promote crystallization of the MET ectodomain through the introduction of a pre-formed twofold axis of symmetry. Size exclusion chromatography, however, suggested the protein to be monomeric. Hence, we used scattering techniques applied to solutions to further investigate its oligomerization state. The small angle X-ray scattering (SAXS) curve measured for our protein nicely fits to the scattering curve calculated from the known crystal structure of a diabody. In addition, concentration-dependent photon correlation spectroscopy (PCS) measurements revealed a hydrodynamic radius of 3.4 nm at infinite dilution and a negative interaction parameter $k_{D}$, indicating attractive interactions that are beneficial for crystallization. Both SAXS and PCS measurements clearly suggest our antibody fragment to be a diabody in solution. Chemical cross-linking with glutaraldehyde and cell motility assays confirmed this conclusion.

## 1. Introduction

Antibodies belong to the most variable proteins of higher organisms. This variability is necessary to recognize a vast number of different pathogens or foreign molecules and to trigger an adequate immune response. There are five different classes of human antibodies (IgA, IgD, IgE, IgG, IgM) with specific tasks [1,2]. IgG is the most abundant antibody in the blood serum and is used for therapeutical purposes. Its overall structure resembles a Y shape with one fragment crystallizable (Fc) and two antigen binding fragments (Fab). The Fc part is responsible for the stability of the antibody and for the recruitment of effector molecules. The antigen binding sites are located at the ends of the Fab arms, in the variable region of the heavy (V${}_{H}$) and the light chains (V${}_{L}$) [3,4].

Antibodies feature highly specific binding to a wide range of antigens. Therefore, antibodies are being increasingly used for therapeutical purposes, such as passive immunization [5,6], immunomodulation (e.g., as therapy for multiple sclerosis) [7,8], cancer therapy (e.g., Trastuzumab [9,10,11] or monomethyl auristatin E [12]) or for medical imaging (e.g., immunoscintigraphy [13,14]). Besides the use of whole antibodies, the application of antibody fragments is widely spread and provides further advantages [15,16]. Antibody fragments can be produced easily by expression in *E. coli* and *P. pastoris* with high yields [17,18]. These fragments can be modified more easily, used as building blocks in biochemical engineering [19,20] and show good pharmaco–kinetic profiles due to their smaller size. They are advantageously usable for drug targeting with the benefit of being capable of penetrating solid tumor tissue quickly as well as showing a fast clearance rate out of the residual tissue [15,16].

**Figure 1:**
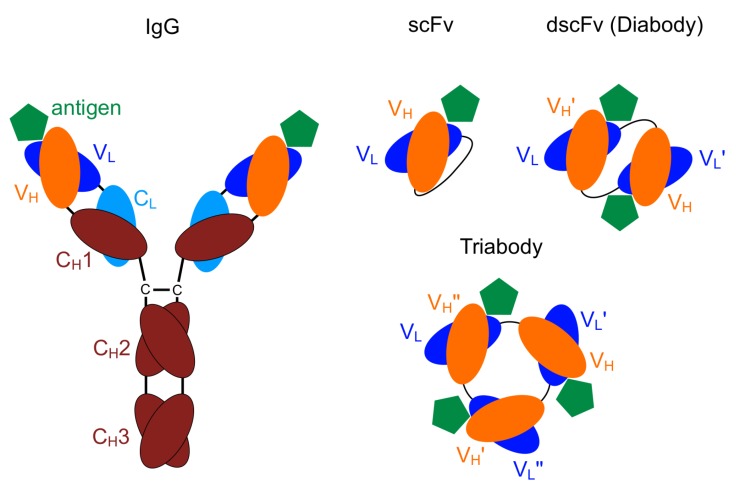
Schematic drawing: immunoglobulin (IgG) including the variable domains, (V${}_{H}$) and (V${}_{L}$), single-chain Fv (scFv), diabody, triabody [23].

The smallest engineered antibody fragment containing the parental specificity is the variable fragment (Fv). This is composed of the variable region of the heavy (V${}_{H}$) and the light chains (V${}_{L}$) [21,22]. Both of these chains can be connected by a linker of a few amino acids resulting in a scFv (Figure 1).

Oligomers of scFv can be obtained by shortening the linker length. A length of less than twelve amino acids leads mainly to the formation of dimers (diabodies), while less then four amino acids results in tri- and tetramers (tria- and tetrabodies) [24,25,26]. Due to the shorter linker length, no binding is possible between the V${}_{H}$ and V${}_{L}$ domains of one chain, therefore the V${}_{H}$ and V${}_{L}$ domains of different chains bind to each other forming oligomers [27,28,29]. Usually there is a strict separation between the formation of different oligomers. Varying the linker length by only one amino acid may shift the equilibrium completely to another oligomeric state with the lowest complexity being most favourable [26].

So far, about 17 crystal structures of diabodies are known [30,31] and several of them use the same base structure. Some of these strutures are of interest in the context of the present work (e.g., [27,32,33]). These show that the two antigen binding sites are located at opposite sides of the protein. Therefore, diabodies are able to bind two antigens or even two different epitopes of the same antigen. This means they are bivalent molecules with a higher avidity than monomeric antibody fragments.

These two binding sites can also be beneficial for crystallization, because of the resulting twofold symmetry [34]. However, according to Kwon et al. diabodies exhibit an important diversity and flexibilty with respect to the orientation of the two scFv units [31]. The diabody (3H3) described in this article binds to the MET receptor, a receptor for tyrosine kinase often involved in the formation of tumors [35] and could therefore be used in cancer therapy. Moreover, the 3H3 diabody might act as crystallization chaperone [36] for the extracellular domain of MET, which has not yet been crystallized in its entirety [37]. However, during the purification, contradictory results about the size of 3H3 were obtained, which indicated that the protein might not be a diabody but rather a scFv, and therefore not suitable for generating the desired twofold symmetry. Another possible explanation for the unexpected size exclusion chromatography (SEC) results might be a strong interaction between the column and the diabody. Hence, to analyze the oligomeric state in solution, different scattering methods were employed to determine the size, shape, molecular mass and hydrodynamic properties of the protein. The used combination of different scattering experiments might be of general interest, since processes such as dimer or oligomer formation can be found for several proteins and oligomers might not stand separation on SEC columns. Other proteins are intriniscally polydisperse like e.g., α-crystallin [38] or might show major structural changes during activation which are not easy to capture by high resolution methods but can be detected in-situ by low resolution approaches as presented here [39,40].

## 2. Materials and Methods

### 2.1. Protein Expression and Purification

*P. pastoris* was stably transfected with the desired target gene and the protein was expressed for five days in minimal medium [41]. The expression was induced by a constant methanol level of 1%. The supernatant was filtered, reduced by cross-flow filtration (membrane: Minimate TFF Capsule, Omega 10 kDa; Pall Corporation, Port Washington, New York) and the buffer was exchanged against phosphate buffered saline (PBS). The protein was then purified by Ni^2+^-NTA affinity chromatography followed by dialysis against the low-salt buffer for ion exchange (IEX) chromatography (50 mM Na-acetate, pH 4.7, 20 mM NaCl). IEX (Source 15S, GE Healthcare, Berlin, Germany) and finally SEC with a HiLoad 16/60 Superdex 75 PG column (GE Healthcare, Berlin, Germany) equilibrated in 20 mM MES, pH 6.5, 150 mM NaCl buffer was used to obtain highly pure protein. The peak fractions of the SEC excluding the long tail were pooled and concentrated to 10 mg·mL^−1^ using Vivaspin ultrafiltration devices. All solution scattering experiments were carried out in 20 mM MES, pH 6.5, 150 mM NaCl with protein diluted from this 10 mg·mL^−1^ stock solution.

### 2.2. Analytical Size Exclusion Chromatography

A Superdex 75 10/300 GL (GE Healthcare, Berlin, Germany) column was equilibrated with 1.3 column volumes of 20 mM MES, pH 6.5, 500 mM NaCl buffer. After injecting the sample, the protein was eluted using 1.3 column volumes during which the UV-absorbance at 280 nm was recorded. The column was calibrated with ferritin (443 kDa, void volume) albumin (66 kDa), ovalbumin (43 kDa), carbonic anhydrase (29 kDa), cytochrome C (12.4 kDa) and aprotinin (6.5 kDa) in PBS right before running the 3H3 concentration range without changing the tubing between column and HPLC system between these runs.

### 2.3. Cell Motility Assay

The ovarian cancer cell line SKOV-3 was grown in RPMI 1640 Glutamax medium (Life Technologies, Monza, Italy), supplemented with antibiotics and 10% (*v*/*v*) fetal bovine serum (Life Technologies). The cell migration assay was performed using a modified Boyden chamber assay (AC96 Migration Chamber; Neuro Probe, Gaithersburg, MD 20877, USA) with a porous membrane (8 μm, PVP-free) previously coated with 100 μg·mL^−1^ Collagen (Purecol, Cellsystems, Troisdorf, Germany) in phosphate buffer saline. Cells were seeded in the top part of the chamber at a density of 106 mL^−1^ resuspended in serum-free RPMI 1640 with 0.25% (*w*/*v*) bovine albumin. 3H3 antibody molecules and HGF/SF were plated in the bottom part of the chamber at selected concentrations. Cell migration was carried on for six hours at 37 °C in a 5 % CO_2_ incubator. After that, the apparatus was disassembled and non-migrated cells on the filter were gently removed by wiping the surface with cotton balls. Migrated cells on the filter were fixed for one hour in 4% formaldehyde and stained for 30 min with DeepRed HCS Cell Mask Stain (Life Technologies) at 1 μg·mL^−1^. Fluorescence intensity was measured using the plate reader Odyssey (LI-COR Biosciences, Lincoln, Nebraska USA) using excitation at 700 nm. Background fluorescence from non-stimulated cells was subtracted and the data shown are the mean of triplicate sample [42].

### 2.4. Small-Angle X-ray Scattering

SAXS measurements were performed using a XEUSS system (XENOCS, Sassenage, France). This provides monochromatic Cu K${}_{\alpha }$ radiation and variable sample to detector distances between 50 cm and 277.5 cm. The setup consists of an evacuated collimation path with two variable scatterless slits providing a beam of squared cross section ( 0.5 mm× 0.5 mm), an evacuated X-ray scattering path and a hybrid-pixel area detector (Pilatus 300k, Dectris, Baden, Switzerland). The protein solutions were placed in a quartz glass tube of 1 mm outer diameter and kept at room temperature. The total measured scattering intensities were corrected by substraction of the scattering intensities of the buffer filled tube and normalized with the time of measurement, the absorption and the concentration of the sample. Glassy carbon was used to bring the data to the absolute scale [43].

For small angle scattering analysis the magnitude of the scattering vector *q* is, in general, given by

(1) $$q=\frac{4\pi n}{\lambda }\sin\left(\frac{\theta }{2}\right),$$

with the refractive index of the medium *n* (1 in the case of X-ray scattering), the wavelength of the incident beam λ and the scattering angle θ.

The scattering intensity I(q) detected by the detector consists of several factors:

(2) $$I\left(q\right)=\frac{N}{V}\Delta b^{2}P\left(q\right)S\left(q\right)=K_{\mathrm{SAXS}}cMP\left(q\right)S\left(q\right).$$

Here N/V is the number density of scatterers, $\Delta b=b_{\mathrm{Protein}}-b_{\mathrm{Buffer}}$ the excess scattering length of the sample, P(q) the form factor representing the protein shape and S(q) the structure factor representing intermolecular interactions. It is assumed that the samples are diluted in a way that no intermolecular interactions occur and S(q)=1. The scattering intensity is also dependent on the concentration *c* of the sample, the molecular mass *M* and on the sample specific optical constant $K_{\mathrm{SAXS}}$.

(3) $$K_{\mathrm{SAXS}}=\frac{N_{A}\Delta b^{2}}{M^{2}}=\frac{{\left(\bar{\nu }\Delta \rho \right)}^{2}}{N_{A}}$$

(4) $$\bar{\nu }=\frac{N_{A}V_{\mathrm{Protein}}}{M}.$$

The optical constant $K_{\mathrm{SAXS}}$ contains information about the specific volume $\bar{\nu }$ of the protein and the excess scattering length density $\Delta \rho =\rho_{\mathrm{Protein}}-\rho_{\mathrm{Buffer}}$.

Guinier analysis was used as one common method for determining the radius of gyration $R_{g}$:

(5) $$I\left(q\right)=I\left(0\right)\exp\left(-\frac{1}{3}R_{g}^{2}q^{2}\right).$$

### 2.5. Photon Correlation Spectroscopy

Photon correlation spectroscopy (PCS) is an established tool to study protein structure in solution [39,44]. In a PCS experiment a digital correlator generates the intensity time correlation function which is subsequently used to compute the electrical field time correlation function $g^{1}\left(t\right)$. In the case of monodisperse samples $g^{1}\left(t\right)$ can be described by a single exponential function.

(6) $$g^{1}\left(t\right)=\exp\left(-\Gamma t\right).$$

The relaxation rate Γ is given by

(7) $$\Gamma =D^{\exp}q^{2},$$

with the mutual translational diffusion coefficient $D^{\exp}$.

However, real samples are polydisperse and might exhibit several relaxation modes stemming for example from aggregates of different sizes. In this case $g^{1}\left(t\right)$ is given by

(8) $$g^{1}\left(t\right)=\int_{0}^{\infty }G\left(\Gamma \right)\exp\left(-\Gamma t\right)d\Gamma ,$$

with G(Γ) being the relaxation rate distribution. G(Γ) can be computed by an inverse Laplace transformation.

For the determination of the z-averaged mutual diffusion coefficient $\langle D^{\exp}\rangle$ the FORTRAN program CONTIN provided by S. Provencher [45] was applied. $\langle D^{\exp}\rangle$ can be related to the hydrodynamic radius $R_{h}$ of an equivalently diffusing sphere using the Stokes–Einstein equation [46]:

(9) $$\langle D^{\exp}\rangle =\frac{k_{B}T}{6\pi \eta R_{h}},$$

with the temperature *T*, the Boltzmann constant $k_{B}$, the viscosity of the solvent η. In the case of an ideal infinitely diluted solution $\langle D^{\exp}\rangle$ corresponds to the self-diffusion coefficient $D^{0}$.

A further parameter which can be obtained from dynamic light scattering experiments is the interaction parameter (dynamical virial coefficient) $k_{D}$ according to:

(10) $$\langle D^{\exp}\rangle =D^{0}\left(1+k_{D}c\right).$$

A positive $k_{D}$ points to repulsive intermolecular interactions, whereas a negative $k_{D}$ indicates attractive interactions [47].

The PCS measurements were performed at two scattering angles, 60${}^{\circ }$ and 135${}^{\circ }$, and with nine different sample concentrations in the range of 2.5 mg·mL${}^{-1}$ to 10 mg·mL${}^{-1}$. The samples with the highest and the lowest concentrations were additionally measured angular dependent in steps of 10${}^{\circ }$.

All samples were prepared directly before the measurements in a laminar flow workbench, centrifuged for 15 min at 21,000× *g* at 4 ${}^{\circ }$C, filtered through a 0.2 μm syringe filter (Cellulose Acetate, VWR International GmbH, Darmstadt, Germany) directly into a cylindrical quartz cuvette (540.110-QS, Hellma GmbH and Co. KG, Darmstadt, Germany) and finally centrifuged for 5 min at 2000× *g* for the removal of dust and air bubbles. The cuvettes were previously cleaned with freshly distilled acetone. All PCS measurements were performed on a 3D photon cross correlation spectroscopy system from LS Instruments (LS Instruments AG, Fribourg, Switzerland).

### 2.6. Static Light Scattering

The 3D photon cross correlation spectroscopy system from LS Instruments in addition was used for statc light scattering experiments. This setup allows the measurement of the scattered intensity in an angular range between 40${}^{\circ }$–130${}^{\circ }$. To obtain the scattering information of the pure sample on an absolute scale and free of influences caused by the setup—called excess Rayleigh ratio $R_{\mathrm{ex}}$—the raw intensity recorded by the detector $I_{\mathrm{raw}}$ has to be corrected angular dependent ($I_{\mathrm{Laser}}$: intensity of the incoming beam, $I_{\mathrm{scattered}}$: corrected scattered intensity):

(11) $$I_{\mathrm{scattered}}=\left(\frac{I_{\mathrm{raw}}\;\cdot \;\sin\theta }{I_{\mathrm{Laser}}}\right),$$

and a normalization with a standard, normally toluene, with a known Rayleigh ratio (scattering intensity on absolute scale) $R_{\mathrm{ref}}$ must be done as described in Equation (12) [48,49]:

(12) $$R_{\mathrm{ex}}=\left(\frac{I_{s}-I_{b}}{I_{\mathrm{ref}}}\right){\left(\frac{n_{b}}{n_{\mathrm{ref}}}\right)}^{2}R_{\mathrm{ref}},$$

with the scattered intensity of the sample $I_{s}$, the buffer $I_{b}$ and the reference $I_{\mathrm{ref}}$, the refractive index of the solvent $n_{b\;}$ and of the reference $n_{\mathrm{ref}}$.

### 2.7. Molecular Mass Determination with SLS—The Zimm-Plot

The data obtained by the static light scattering experiments [50] were evaluated with the Zimm Equation (13).

This equation relates the form factor P(θ) to the optical constant *K*, the mass concentration *c*, the molecular mass *M* and the second osmotic virial coefficient $A_{2}$.

(13) $$\frac{Kc}{R_{\mathrm{ex}}}=P{\left(\theta \right)}^{-1}\left(\frac{1}{M}+2A_{2}c\right).$$

When using the Guinier approximation and a simplification, Equation (13) can be written as:

(14) $$\frac{Kc}{R_{\mathrm{ex}}}=\frac{1}{M}\left(1+\frac{q^{2}}{3}\langle R_{g}^{2}\rangle \right)+2A_{2}c,$$

with

(15) $$K=\frac{4\pi^{2}\;\cdot \;n^{2}}{N_{A}\;\cdot \;\lambda_{0}^{4}}\;\cdot \;{\left(\frac{dn}{dc}\right)}^{2},$$

containing the radius of gyration $\langle R_{g}\rangle$, the Avogadro constant $N_{A}$, the wavelength of the light in vacuo $\lambda_{0}$ and the refractive index increment dn/dc.

For the construction of the Zimm diagram $\frac{Kc}{R_{\mathrm{ex}}}$ is plotted against $q^{2}+kc$ and an extrapolation to q=0 and c=0 is done. On the basis of these extrapolations the molecular mass, the second virial coefficient, the radius of gyration is yielded. The scaling factor *k* only influences the appearance of the plot. Hence, the Zimm equation and the plot consolidate all experimental and molecular parameters of the static light scattering experiment.

All measurements were done at 20 ${}^{\circ }C$ and the scattered intensities of the samples were recorded in the scattering angle range between 40${}^{\circ }$ and 130${}^{\circ }$ in steps of 1${}^{\circ }$. For the measurements of the buffer solution and the toluene reference the same parameters were used. All samples were prepared analogously to those used in PCS measurements.

### 2.8. Density, Refractive Index and Refractive Index Increment Measurement

The density and the refractive index measurements were performed on a DMA 4500 density meter and on an RXA 170 refractometer from Anton Paar (Graz, Austria). All measurements were repeated at least three times and the averaged values were taken for further calculations. The refractive index increment was determined using the α-Ref.-System (Version 2.1) from SLS-Systemtechnik (Denzlingen, Germany) with a quartz cuvette (410.45, Starna GmbH, Pfungstadt, Germany). All measurements were performed at 20 ${}^{\circ }C$.

### 2.9. Molecular Mass Calculation from SAXS Data

Determining the molecular mass was an important step to be able to identify the degree of oligomerization of the protein. The molecular mass can be determined using scattering data on absolute scale by extrapolation to I(0) [51] and using the following formula [52]:

(16) $$M_{\mathrm{Prot}}=\frac{I{\left(0\right)}_{\mathrm{abs}}N_{A}}{c_{\mathrm{Prot}}{\left(\Delta \rho \nu \right)}^{2}}.$$

Another approach is to compare the scattering data of the sample with the scattering data of a standard [53] e.g., lysozyme. The molecular mass can then be determined by:

(17) $$M_{\mathrm{Prot}}=\frac{I{\left(0\right)}_{\mathrm{Prot}}}{c_{\mathrm{Prot}}}\frac{M_{\mathrm{Lys}}}{I{\left(0\right)}_{\mathrm{Lys}}/c_{\mathrm{Lys}}}.$$

### 2.10. Prediction and Evaluation of a Possible Structure Model

To obtain a first idea of what kind of oligomer the 3H3 forms, we simulated the scattering curves of an scFv (1X9Q [54]), a diabody (1LMK [27]) and a triabody (1NQB [55]). For this purpose known crystal structures of homologous proteins from the RCSB protein data bank (PDB) were used and their scattering intensities were calculated using the software CRYSOL [56] from the ATSAS software package. With the best fitting structure from the CRYSOL calculation hydrodynamic calculations were performed with the program HYDROPRO [57,58,59,60]. HYDROPRO allows to compute the theoretical diffusion coefficient of proteins based on a structural model [61,62].

## 3. Results and Discussion

### 3.1. SEC Analysis

**Figure 2:**
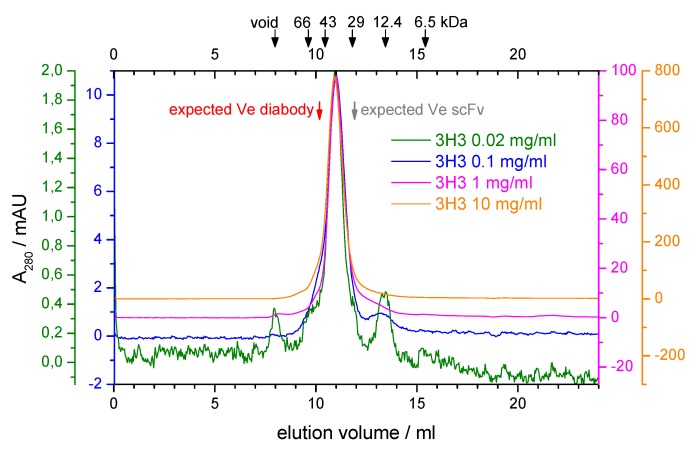
Analytical size exclusion chromatography of 3H3. A Superdex 75 10/300 column was equilibrated with 20 mM MES, pH 6.5, 500 mM NaCl. Four runs with 100 μL of 3H3 at the indicated protein concentrations were performed. The absorbance at 280 nm (A${}_{280}$) is shown with a separately scaled *y*-axis for each concentration so that the baselines are slightly offset and the maxima coincide, thus allowing accurate comparison of elution volumes and peak profiles. The elution volume of standard proteins in a dedicated calibration run is indicated by arrows at the top. The void volume was determined with ferritin (443 kDa). The expected elution volume (Ve) for the diabody ( 54 kDa) and the scFv (27 kDa) were calculated based on the calibration and are indicated with red and gray arrows, respectively.

During purification of our 3H3 construct there were hints that it might not form a diabody. The protein eluted with a main peak corresponding to a molecular mass of around 33 kDa from a preparative SEC run at high protein concentration ( 10 mg·mL${}^{-1}$) but low salt concentration (20 mM NaCl) (Figure S1A, see Supporting Information). The long tail was not due to protein degradation as shown by a SDS-PAGE analysis (Figure S1B), possibly indicating protein adsorption to the column matrix. We analyzed the oligomeric state of the protein further by analytical SEC. At low protein concentration there was no elution peak at low and physiological salt concentrations (20 mM and 150 mM NaCl) again indicating potential adsorption of the protein to the column matrix, while at higher salt concentrations (500 mM and 1 M NaCl) peaks of increasing height appeared at a constant elution volume (data not shown). We performed SEC runs at 500 mM NaCl covering a 500-fold range of protein concentrations, reaching from the detection limit of our UV detector ( 20 μg · mL${}^{-1}$) to the highest protein concentration that we could afford ( 10 mg·mL${}^{-1}$). The elution volume stayed constant over the whole concentration range and corresponded to a molecular mass of 41 kDa (Figure 2). This is in between the calculated molecular mass of the scFv (27 kDa) and that of the diabody (54 kDa). Only an elution volume larger than expected can be explained fairly easily by a non-globular shape of the sample. Based on the SEC results one might, therefore, conclude that no diabody is formed. However, crosslinking experiments indicated that the 3H3 is a dimeric protein (see Figure S2 in the Supporting Information).

Some single-chain Fvs show a dynamic, concentration-dependent equilibrium between monomer and dimer. In one report, two well-separated peaks corresponding to the monomer and the dimer (diabody) were observed in SEC runs with the scFv MFE-23 [63]. At low protein concentration (0.5 mg·mL${}^{-1}$) the dimer peak was barely visible, while at 10 mg·mL${}^{-1}$ the dimer peak almost reached the same height as the monomer peak. We did not observe such a double peak on SEC over a 25-fold wider concentration range (20 μg·mL${}^{-1}$–10 mg·mL${}^{-1}$) suggesting that the unusual elution behavior of the 3H3 diabody is caused by an interaction between the protein and the SEC matrix. It also seems unlikely that our 3H3 diabody could form a monomeric scFv because of its short linker length. Our 3H3 diabody contains a four-residue Gly${}_{4}$ linker, while the MFE-23 scFv that showed reversible dimer formation had a 15-residue (Gly${}_{4}$Ser)${}_{3}$ linker [63]. In addition, we also prepared a covalently linked dimer of two Fab fragments from 3H3 by digesting the intact antibody with pepsin. This F(ab’)${}_{2}$ fragment eluted from a Superose 12 10/300 column as a single, monomeric peak with a calculated molecular weight of about 60 kDa (data not shown), which again is closer to the molecular weight of a monomeric Fab fragment (50 kDa) than to the actual molecular weight of the dimeric F(ab’)${}_{2}$ fragment (100 kDa). Due to the covalent linkage of the two Fab fragments via a disulfide bridge, a monomer–dimer equilibrium cannot be at play here. In this case the bead chemistry of the column is somewhat different from the Superdex matrix used in our other experiments. Hence, SEC on 3H3 and its fragments seems to lead to unexpected results.

To resolve the discrepancy we used solution scattering methods to investigate the protein in its native state. The combination of PCS and SAXS provides complementary information on the overall size, the molecular mass and the low resolution structure of proteins.

### 3.2. SAXS Data Analysis

**Figure 3:**
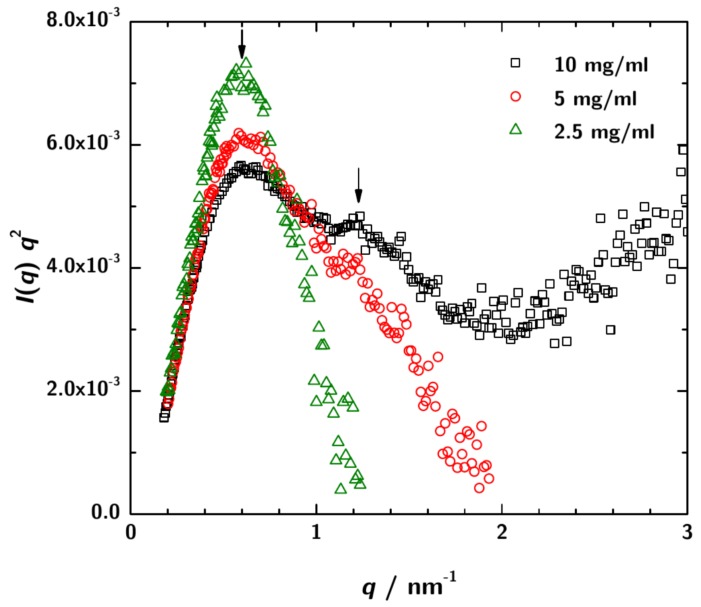
Kratky-plot of the calibrated SAXS data over the total measured *q*-range. For the lowest concentration ( 2.5 mg·mL${}^{-1}$) the data are rather noisy, especially at high *q*. However, for 3H3 concentrations 5 mg·mL${}^{-1}$ and 10 mg·mL${}^{-1}$ a shoulder is obsered in the plots indicating the multidomain character of the protein in solution. This is the expected pattern for a diabody.

SAXS measurements were performed with sample concentrations of 2.5 mg·mL${}^{-1}$, 5 mg·mL${}^{-1}$ and 10 mg·mL${}^{-1}$ at three sample-to-detector distances to cover a possible *q*-range from 0.04 /nm to 6.78 /nm. Figure 3 shows a Kratky-plot of the experimental data.

For the lowest 3H3 concentration the data at high *q* are rather noisy. However, for the higher concentrations the typical scattering pattern for a multidomain protein is observed.

**Figure 4:**
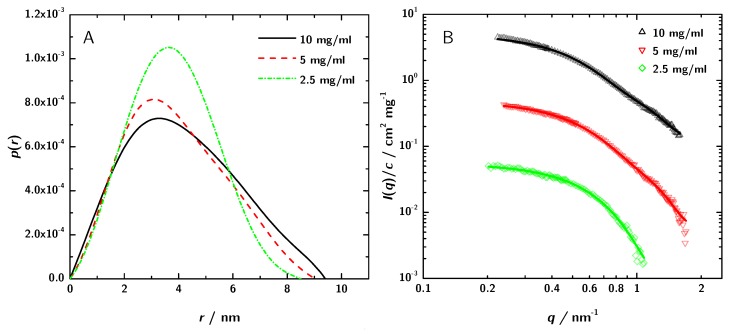
(**A**) p(r)—functions obtained by GNOM and (**B**) the corresponding fit in the reciprocal space (for clarity, the upper two data sets have been shifted by a factor of 10 and 100, respectively).

The usable q-range after buffer subtraction ranges from 0.18 nm^−1^ to 1.67 nm^−1^ (Figure 4B). The plot of the concentration normalized data shows no major difference in the scattering intensities in the q-region between 0.3 nm^−1^ and 1 nm^−1^. Therefore a significant concentration dependent change of the shape can be ruled out. The decrease in intensity of the concentration normalized scattering curves at low q-values with increasing sample concentration can be explained by the contribution of the structure factor, which at least at 10 mg·mL${}^{-1}$ does not strictly follow S(q)≈1. The PCS experiments which are discussed in Section 3.4 indicate attractive interaction. According to Tardieu et al. [64] this would mean S(q=0)>1. The shape of the black curve in Figure 4B might also indicate this. However, the influence of S(q) is only marginal in the scrutinized *q*-range.

The structural parameters and the molecular mass of the 3H3 antibody fragment derived from these curves are given in Table 1. The radius of gyration $R_{g}$ estimated by the Guinier approximation Equation (5) is 3.36±0.09 nm. The GNOM calculation yields $R_{g}=$ 3.06 nm. GNOM was also used to calculate the pair distance distribution function p(r) (Figure 4A).

**Figure 5:**
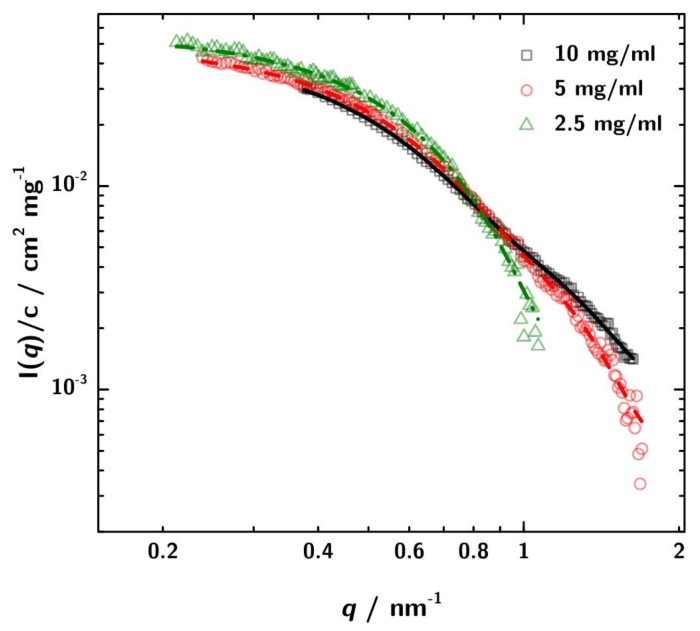
SAXS data normalized to concentration with the respective GNOM fits. The discrepancies between the curves can be attributed to increased interaction between the 3H3 molecules at higher concentration. These differences lead to a variation in the obtained apparent radius of gyration $R_{g}$ of about ±5 to 6%.

For better comparison in Figure 5 the SAXS data are normalized to the different protein concentration. In addition, also the respective GNOM fits are shown.

The differences in the curves can be attributed to stronger interaction at higher concentration and lead to minor differences between the computed $R_{g}$ values. However, for the conclusions derived later on, the differences are not relevant.

The molecular mass of the protein can be determined from the scattering data in several ways. By extrapolation to I(0) a mean molecular mass of 70.8 kDa was obtained. The comparison with lysozyme as standard resulted in a mean molecular mass of 46.5 kDa (Table 1). Compared to the calculated molecular mass of the diabody of 53.9 kDa the results deviate in the range of 9% to 41%, compared to the molecular mass of the scFv with 26.95 kDa the deviation is much higher (61% to 183%). The molecular mass determination by SAXS leads to the assumption that 3H3 is actually a dimer. To reinforce this result, simulations were done.

### 3.3. Calculation of SAXS Data with CRYSOL

**Figure 6:**
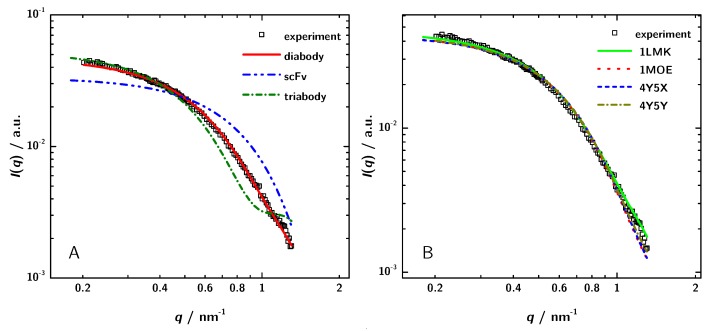
(**A**) Comparison of simulated scattering patterns of a monomeric scFv, a diabody and a triabody computed with CRYSOL to experimental data. (**B**) Comparison of theoretically calculated scattering paterns of several different diabodies with the experimental data. The scattering patterns of the different diabodies were obtained using the PDB entries and CRYSOL. The shown experimental data were obtained for the sample with a concentration of 5 mg·mL${}^{-1}$.

To verify the conclusion based on the different determinations of the molecular mass and to reveal the structure of 3H3 in solution in an independent way, the theoretical scattering curves of the different possible oligomers were calculated by CRYSOL [56] and compared with the experimental data. The calculations were based on a choice of crystal structures of homologous proteins listed in the PDB. Coordinates from PDB entries 1X9Q [54], 1LMK [27] and 1NQB [55] were used to calculate theoretical scattering curves of a monomeric scFv, a diabody and a triabody, respectively (Figure 6). Figure 6 shows the experimental data for the 3H3 protein solution at a concentration of 5 mg·mL${}^{-1}$ and the results from the simulated scattering curves. The calculated curve of the diabody structure nicely fit to the measured data, whereas both of the other curves show large deviations. Hence, the model calculations clearly show that 3H3 forms a diabody in solution.

As a next step, the experimental scattering patterns were compared with the calculated scattering data of diabody structures from the PDB (1LMK, 1MOE, 4Y5X, 4Y5Y) to identify the structurally most similar model for 3H3. Figure 6 shows that all the calculated curves almost fit to the measured data. The calculated scattering data of 1LMK matches the experimental data best with a chi${}^{2}$-value of 1.60.

### 3.4. Photon Correlation Spectroscopy (PCS) Data

**Figure 7:**
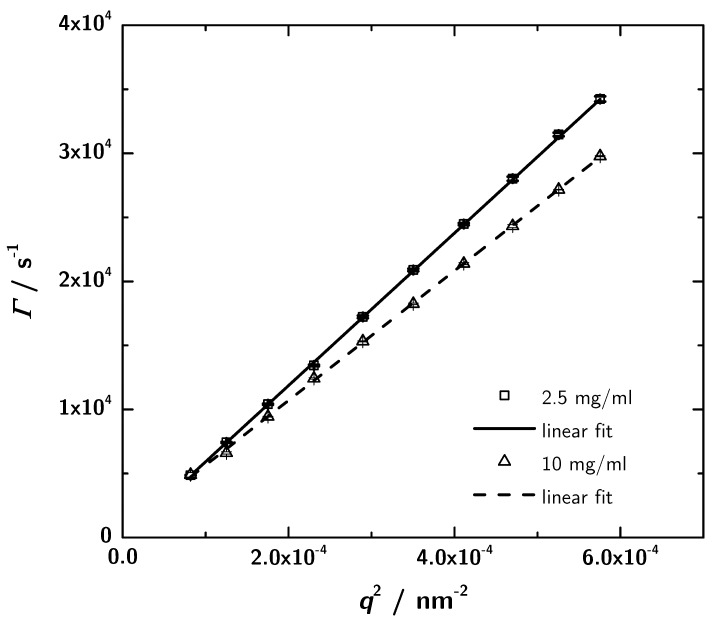
Plots of the relaxation rate Γ vs. $q^{2}$ for 2.5 mg·mL${}^{-1}$ and 10 mg·mL${}^{-1}$. The slope yields the translational diffusion coefficient (see Table 2). Within the experimental precision the intercept is zero, as expected for the simple center of mass diffusion.

PCS provides another independent way to determine protein structures in solution in a rather fast and easy way. For the determination of the hydrodynamic properties of the 3H3 diabody photon correlation spectroscopy measurements were performed at different sample concentrations and two scattering angles. For two concentrations (the lowest and the highest) angular-dependent measurements were performed. The obtained data were analyzed using CONTIN. The relaxation rate distribution shows one sharp peak and at lower angles a broad contribution which indicates a small amount of aggregates. Plotting the relaxation rate of the main mode against the squared scattering vector (Figure 7) leads to the translational diffusion coefficient $D^{\exp}$ (Table 2) according to Equation (7). The interaction parameter $k_{D}$ is given by the slope of a concentration-dependent plot of the translational diffusion coefficient (Equation (10); data not shown) and is determined to $-1.50\times 10{}^{-2}$ mg·mL${}^{-1}$ from the measurements at a scattering angle of 60${}^{\circ }$. Negative values stand for attractive intermolecular interactions between protein molecules in the given solvent [47], which denotes a good precondition for crystallization. Analyzing the translational diffusion coefficient regarding the measurements at different sample concentrations, the extrapolation to zero concentration results in the self diffusion coefficient $D^{0}$ = $6.295\times 10{}^{-11}$ $m^{2}$·s${}^{-1}$ and the associated $R_{h,0}$ = 3.4 nm. The self diffusion coefficient describes the diffusion behaviour of a single molecule.

### 3.5. Calculation of Hydrodynamic Data with HYDROPRO

In a further evaluation step the hydrodynamic properties of the scFv structure 1X9Q and of the similar diabody 1LMK were calculated using the software HYDROPRO [57,58,59,60]. The temperature, the solvent viscosity η and density ρ, the partial specific volume $\bar{\nu }$ and the molecular mass *M* were set to the values in Table 3, for the other parameters default values of HYDROPRO were used. The hydrodynamic radius was calculated from the results using Equation (9).

$D^{0}$ calculated for the structural model of a diabody was $5.94\times 10{}^{-11}$ $m^{2}$·s${}^{-1}$. Hence, within the experimental error an excellent agreement between the PCS experiment and the model calculation of this diabody is obtained.

### 3.6. Zimm Analysis

**Figure 8:**
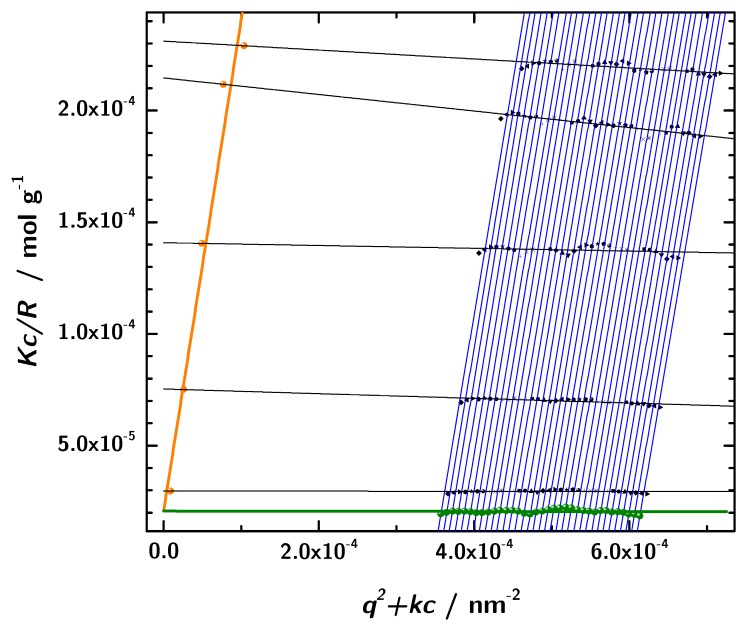
The Zimm-diagram obtained from the static light scattering measurements (black: measured data, orange: extrapolation to q=0, green: extrapolation to c=0, lines: linear fits).

In addition to the determination of hydrodynamic properties using photon correlation spectroscopy, static light scattering in combination with the Zimm analysis was used to obtain the molecular mass. The basis for this analysis was the data obtained from angular dependent static light scattering measurements of samples with five different protein concentrations. The formalism is based on the assumption of linear dependence of the intensity on concentration and *q*, respectively. This approach allows us to determine the molar mass and other parameters at zero protein concentration [49]. In a broad range of scattering angles and concentrations the experimental data showed the expected linear behavior and consequently the Zimm formalism can be applied to these linear regions. In Figure 8 the results with a linear fit and the extrapolation according to the Equations (13) and (14). The intercept of both extrapolated lines with the *y*-axis is at $2.0666\times 10{}^{-5}$ mol·g${}^{-1}$. Thus, the resulting molecular mass obtained by static light scattering is 48.4 kDa (data listed in Table 4. This is in very good accordance with the sequence based molecular mass of 53.9 kDa of the 3H3 diabody. Moreover, this rules out any significant contribution from unimers.

### 3.7. Cell Motility Assay

**Figure 9:**
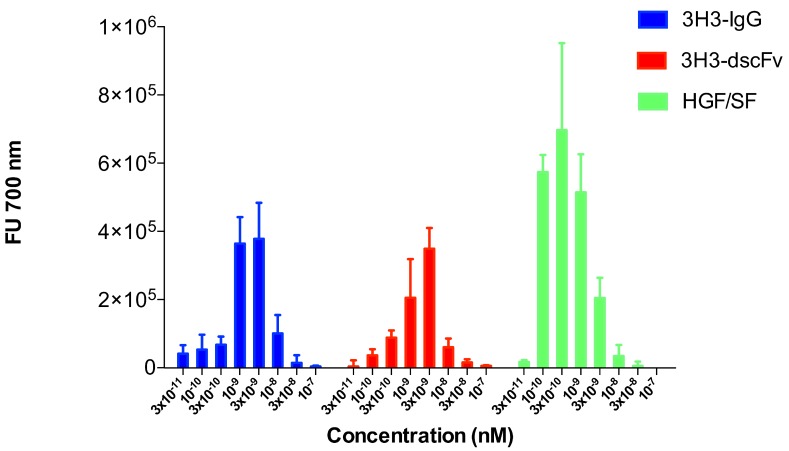
Cell motility assay (migration of SKOV-3 cells). A modified Boyden chamber assay was used to analyze stimulation of cell motility in SKOV-3 cells. HGF/SF served as positive control and showed the typical bell-shaped dose-response curve with activity down to 100 pM. The intact 3H3 antibody (3H3-IgG) and the 3H3 diabody (3H3-dscFv) also showed a bell-shaped dose-response curve. The diabody stimulated cell migration down to a concentration of 100 pM.

The scattering experiments were performed at relatively high protein concentrations of 1 to 10 mg/mL. We used a sensitive cell motility assay to obtain hints about the oligomeric state of the 3H3 diabody at very low protein concentration. Cell motility is a result of MET activation, which in turn is a caused by MET dimerization. The endogenous MET ligand HGF/SF served as positive control and stimulated migration of SKOV-3 cells at a concentration as low as 100 pM (Figure 9). The 3H3 diabody showed a bell-shaped dose-response curve very similar to that of the intact 3H3 antibody (Figure 9). Both showed maximal activity at 3 nM (160 ng·mL${}^{-1}$), while at 100 nM no stimulation of cell migration was observed. The 3H3 diabody was active down to a concentration of 300 pM (16 ng·mL${}^{-1}$). This strongly suggests that our 3H3 construct is bivalent like the intact 3H3 IgG molecule and thus is dimeric even at low concentration.

## 4. Conclusions

In this work we have studied the solution structure of the IgG fragment 3H3. Contrary to the results obtained by SEC, indicating the presence of only unimers in solution, it could be clearly shown by non-invasive low resolution scattering experiments that in fact predominantly diabodies are formed in solution. This assumption is supported by the Zimm analysis of 3H3 that at infinite dilution yielded a hydrodynamic radius in good accordance with the predicted molecular mass of the diabody (see Section 3.6). Using the structure of 1LMK as a model for a diabody in hydrodynamic model calculations based on the methods introduced by Garcia de la Torre, we calculated a translational diffusion coefficient of $5.94\times 10{}^{-11}$ $m^{2}$·s${}^{-1}$. This is in good agreement with the experimental value of $D^{0}=$ $6.295\times 10{}^{-11}$ $m^{2}$·s${}^{-1}$. In conclusion, our experiments convincingly show 3H3 can produce the desired twofold symmetry that is beneficial for crystallization. Finally, the bell-shaped dose-response curve in the cell motility assay strongly suggests that our 3H3 construct actually forms a bivalent diabody able to dimerize MET at a concentration as low as 1 nM (≈50 ng mL${}^{-1}$). Hence, trying to use the 3H3 diabody as a crystallization chaperone in complex with the MET receptor seems a promising approach.

## Figures and Tables

**Table 1:** Structural parameters derived from experimental SAXS data. Here, $R_{g}$ indicates the radii of gyration as computed by a Guinier and GNOM analysis, respectively. $D_{max}$ is the maximum dimension of the 3H3 diabody is obtained from the p(r) functions. M(lys) is the molecular mass as obtained from the SAXS data using lysozyme as a standard.

Parameter		Experiment		1LMK
	10 mg·mL^−1^	5 mg·mL^−1^	2.5 mg·mL^−1^	
$R_{g}$ (Guinier)/nm	3.66	3.29	3.27	2.9
$R_{g}$ (GNOM)/nm	3.24	3.04	2.89	
*D*_max_/nm	9.4	9.02	8.50
M(lys)/kDa	46.8	43.4	49.3	

**Table 2:** Hydrodynamic parameters measured by photon correlation spectroscopy (PCS) (PCS is often also called DLS (dynamic light scattering). $D^{exp}$ is the experimentally found translational diffusion coefficent as obtained from a numerical analysis of the PCS data. The hydrodynamic radius $R_{h}$ is computed on the basis of $D^{exp}$ (see the Materials and Methods section).

Parameter	2.5 mg·mL^−1^	10 mg·mL^−1^
*D*^exp^/nm^2^·s^−1^	5.95 × 10^7^	5.06 × 10^7^
$R_{h,\exp}$/nm	3.60	4.24

**Table 3:** Calculated hydrodynamic properties, $R_{\mathrm{element}}$ consists of 2.9 Å (recommended by the software) for one element and 3 Å for the first hydration shell, the calculation is based on the: atomic level primary model, shell calculation (type 1). The listed experimental values are extrapolations for infinite dilution. Here, $\bar{\nu }$ is the specific average volume of a protein, ρ and η are the density and the viscosity of the buffer.

	1X9Q (scFv)	1LMK (Diabody)	Experiment
$R_{\mathrm{element}}$/Å	5.9	5.9	-
Type of calculation	1	1	-
T/°C	20	20	20
η/mPa s	101.6	101.6	101.6
M/kDa	29.447	53.911	-
$\bar{\nu }$/cm^3^g^−1^	0.725	0.731	-
ρ/g cm^−3^	1.00677	1.00677	-
$D^{0,\mathrm{theo}}$/m^2^·s^−1^	7.74 × $10^{-11}$	5.94 × $10^{-11}$	6.295 × $10^{-11}$
$R_{g,\mathrm{theo}}$/ nm	2.1	2.9	-
$R_{h,0,\mathrm{theo}}$/ nm	2.8	3.6	3.4
$R_{g,\mathrm{theo}}/R_{h,\mathrm{theo}}$	0.75	0.81	-

**Table 4:** Parameters used for the Zimm-analysis. The listed parameters are input values for the analysis and are defined in the Materials and Methods section. The given refractive index increment dn/dc results from measurements on our 3H3 samples and is about 25 % larger compared to values reported in the literature [65].

Parameter	Value
*c*/ mg·mL${}^{-1}$	0.91, 2.58, 4.9, 7.71, 10.0
$R_{\mathrm{ex},\;\mathrm{Toluene}}$/cm^−1^	13.43 × 10^−6^
dn/dc/cm^3^g^−1^	0.267
$n_{b,}\;\mathrm{MES}-\mathrm{Buffer}$	1.335
$n_{\mathrm{ref},}\;\mathrm{Toluene}$	1.496
*k*/ nm·g${}^{-1}$	10
*K*/mol (m/g)^2^	5.55038 × 10^−11^

## Supplementary Materials

The following are available online at https://www.mdpi.com/2073-4468/8/4/48/s1, Figure S1: Preparative size exclusion chromatography of the 3H3 diabody; Figure S2: Analysis of chemical crosslinking with glutaraldehyde; Figure S3: Distribution of the relaxation rates as computed by a CONTIN analysis. In the respective photon correlation spectroscopy experiment a 3H3 solution with a concentration of 10 mg·mL^−1^ was measured at two scattering angles (60° (A); 100° (B)); Figure S4: Distribution of the relaxation rates as computed by a CONTIN analysis. In the respective photon correlation spectroscopy experiment a 3H3 solution with a concentration of 2.5 mg·mL^−1^ was measured at two scattering angles (60° (A); 100° (B)).
