# Supplementary material for: Distinguishing Between Monomeric scFv and Diabody in Solution Using Light and Small Angle X-ray Scattering
Source: Antibodies (Basel). 2019 Sep 23;8(4):48. doi: 10.3390/antib8040048 (PMC6963988; doi:10.3390/antib8040048)
Supplement: Supplementary file 1 [file antibodies-08-00048-s001.pdf]

Distinguishing between monomeric scFv and diabody using light and small angle x-ray scattering

F. Lüdel, S. Bufe, W. M. Bleymüller, H. de Jonge, L. Iamele, Hartmut H. Niemann, T. Hellweg*

Supporting Material

Addtional SEC Experiment

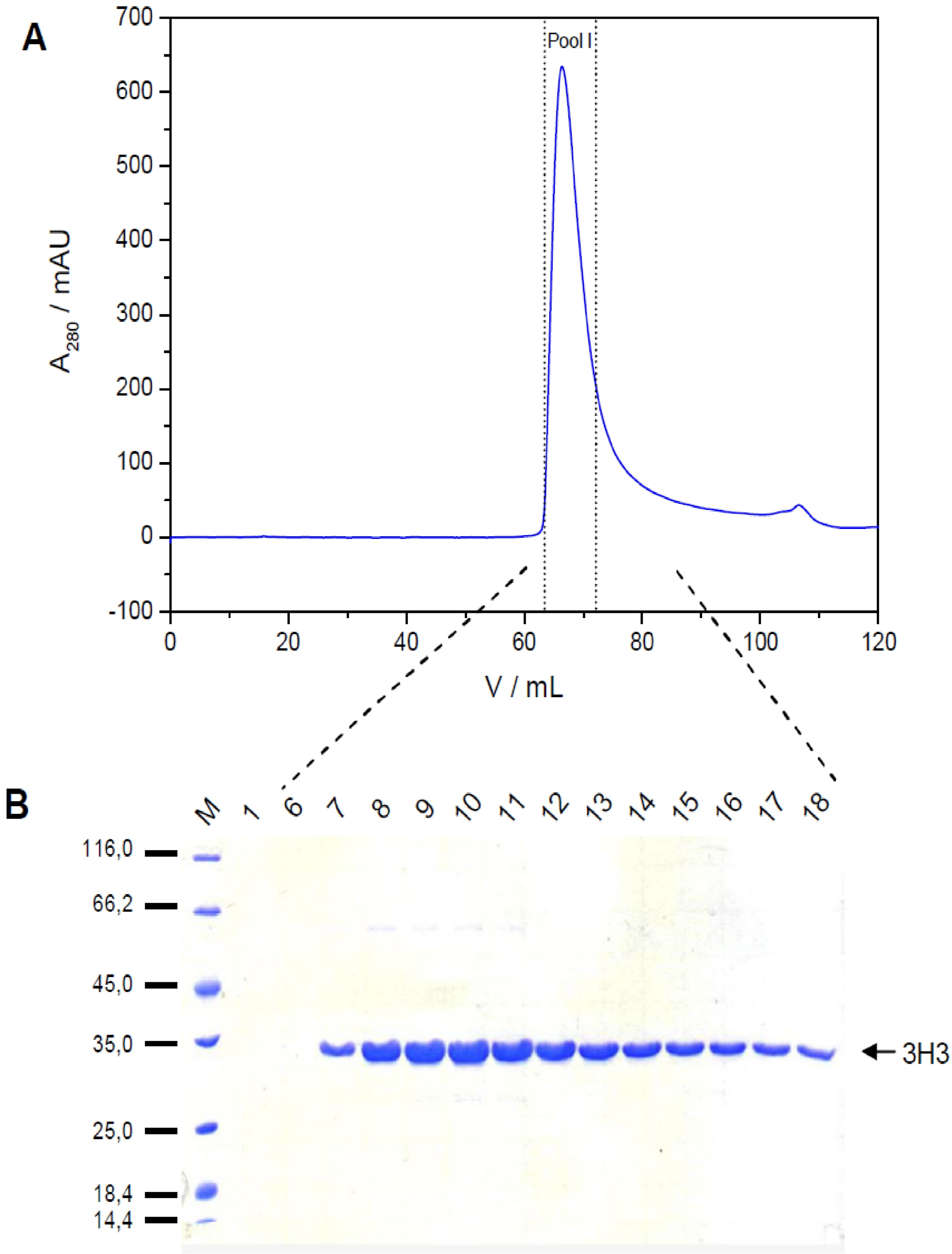

Figure S1: Preparative size exclusion chromatography of the 3H3 diabody. 4.3 mg of 3H3 were loaded on a HiLoad 16/60 Superdex 75 PG column equilibrated in 20 mM MES, pH 6.5, 20 mM NaCl. (A) Chromatogram of a preparative SEC run. The elution volume of the peak around 66 ml corresponds to a molecular mass of around 33 kDa. The

pooled fractions are indicated by dotted vertical lines and labelled ”Pool I”. (B) Reducing SDS-PAGE of the fractions indicated by dotted lines revealed that the tailing is not due to protein degradation. Only intact 3H3 could be detected over the entire elution volume.

Chemical Crosslinking

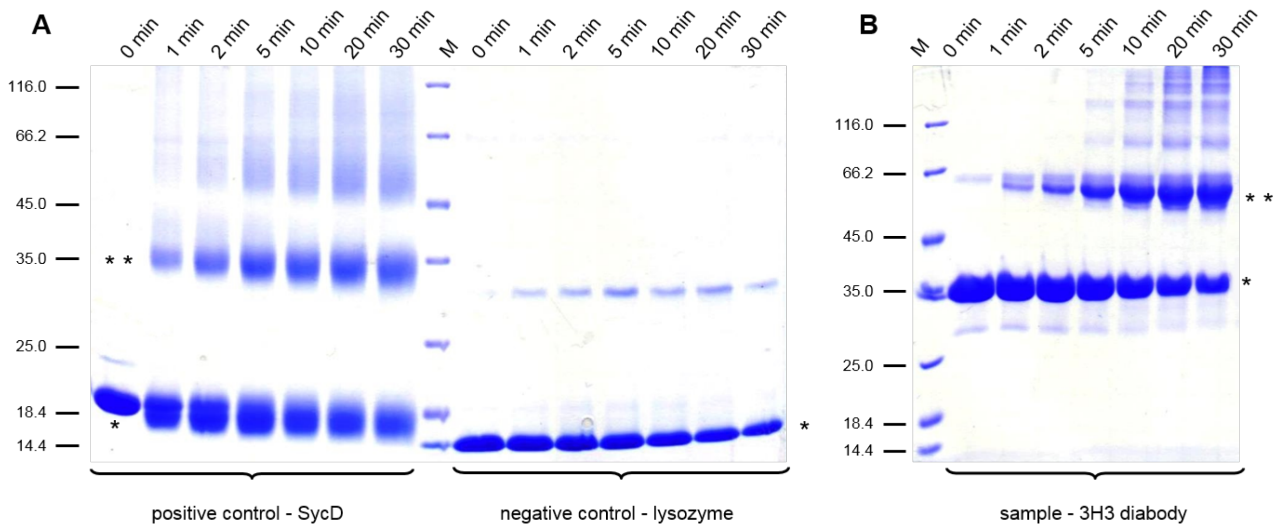

Figure S2: Analysis of chemical crosslinking with glutaraldehyde. 100 µl of protein solution (1 mg/ml) was mixed with 5 µl 2.5 % glutaraldehyde. Samples were taken at the time stated in the figure, the reaction was stopped by adding 1 M tris pH 8 and the sample was analysed by SDS-PAGE (12% gels). 10 µg of protein were loaded per lane and the gels were stained with Coomassie solution. (A) positive control SycD21-163 (previously shown to form dimers, [55]), negative control lysozyme in 1 x PBS pH 7.4. The positive control shows a band in the region of the dimer (**) that increased with time while that of the monomer is decreasing in intensity. The band in the region of the dimer that can be seen in the negative control can be neglected due to its comparably low and constant intensity. (B) 3H3 diabody purified by SEC in 20 mM MES with 20 mM NaCl pH 6.5. Due to the crosslinking a band at the size of the dimer can be seen comparable to the positive control.

Relaxation Rate Distribution obtained from CONTIN

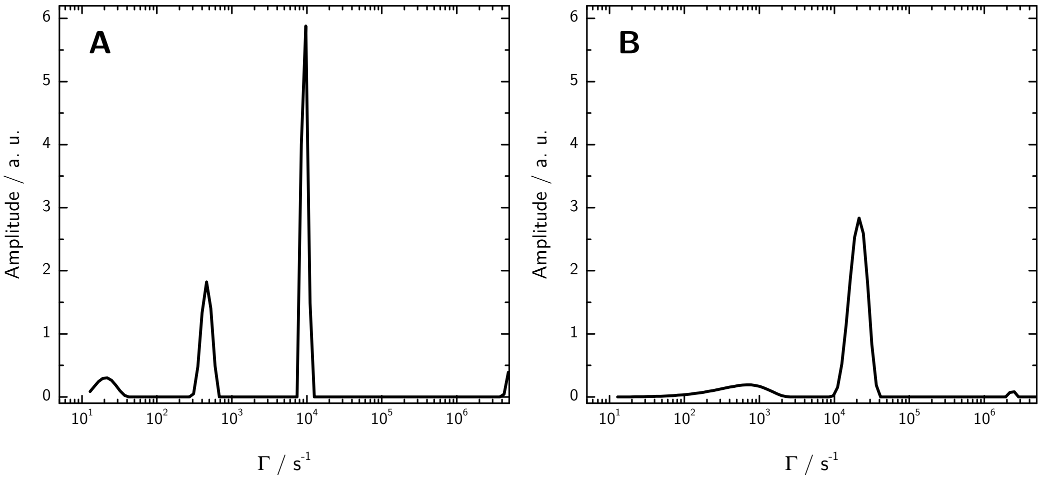

Figure S3: Distribution of the relaxation rates as computed by a CONTIN analysis. In the respective photon correlation spectroscopy experiment a 3H3 solution with a concentration of $10\ \mathrm{mg/ml}$ was measured at two scattering angles (60° (A); 100° (B)). The measurements were performed at 20 °C. For the determination of the diffusion coefficient the peak with the largest integral was used. Peaks at lower frequencies correspond to minority species (dust or aggregates), the high frequency peak is a numerical artefact often observed in the CONTIN analysis data.

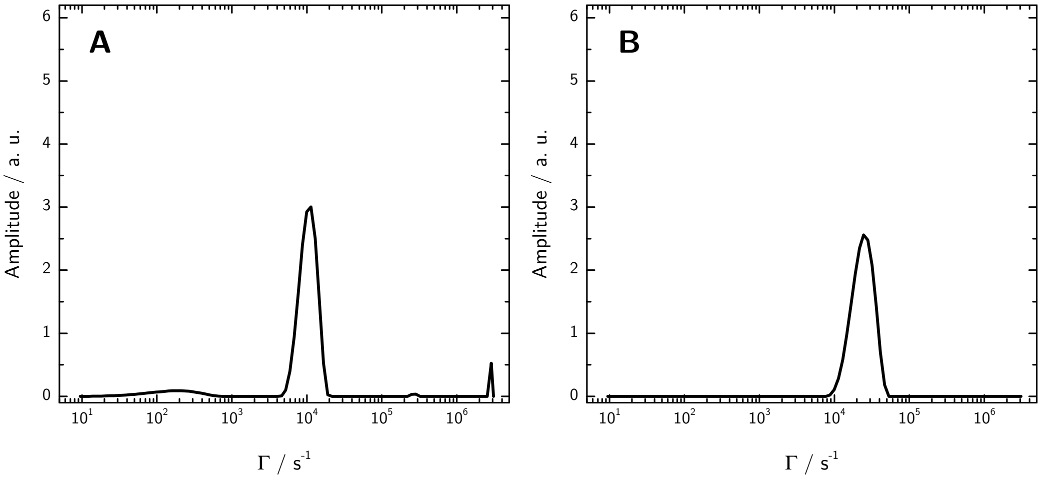

Figure S4: Distribution of the relaxation rates as computed by a CONTIN analysis. In the respective photon correlation spectroscopy experiment a 3H3 solution with a concentration of $2.5\ \mathrm{mg/ml}$ was measured at two scattering angles (60° (A); 100° (B)). The measurements were performed at 20 °C. For the determination of the diffusion coefficient the peak with the largest integral was used. Peaks at lower frequencies correspond to minority species (dust or aggregates), the high frequency peak is a numerical artefact often observed in the CONTIN analysis data.
